# Physical Activity Counseling in Saudi Arabia: A Systematic Review of Content, Outcomes, and Barriers

**DOI:** 10.3390/ijerph192316350

**Published:** 2022-12-06

**Authors:** Mezna A. AlMarzooqi, Franziska Saller

**Affiliations:** 1 Department of Community Health Sciences, College of Applied Medical Science, King Saud University, Riyadh 11451, Saudi Arabia; 2 Department of Project Management, Universidad Internacional Iberoamericana, Campeche 24560, Mexico

**Keywords:** physical activity, physical activity counseling, counseling standards, perceived barriers, Saudi Arabia

## Abstract

Objectives: This study aimed to map the characteristics and the predominant components of clinical physical activity (PA) counseling in Saudi Arabia for adult patients and outline evidence of outcomes and prevalent barriers to its implementation. Methods: We conducted a systematic literature search of four online databases: Web of Science, PubMed, ScienceDirect, and The Cochrane Library. Each study was assessed and evaluated using the Mixed Methods Appraisal Tool (MMAT) for methodological quality. Results: A total of 120 studies were screened, and 47 studies were sought for retrieval. In total, 25 articles were eligible and were subjected to extensive review. After a detailed evaluation, only nine studies met the inclusion criteria. All included were quantitative studies that compiled descriptive and numerical data on physical activity counseling. Four studies described PA counseling information in Saudi Arabia or prescription as lifestyle modification and program structure. The programs used various techniques to motivate patients to adhere to PA protocols. In general, practitioners indicated a high perceived competence in helping patients meet PA guidelines. The most frequently stated barrier was a lack of time for PA discussions with patients, followed by a lack of training in PA counseling, and a lack of patient compliance. Significant improvements in clinical parameters and smoking, food, and exercise habits were detected in experimental trials with respective intervention programs. Conclusion: This review provides preliminary insights into the delivered intervention and standard care content, its outcomes, and clinicians’ perceived competence and barriers regarding current PA counseling approaches in Saudi Arabia. Despite the small number of studies included, this review contributes to the limited understanding of current PA counseling practices in Saudi Arabia and serves as an informational source for clinicians and policymakers and a starting point for further research.

## 1. Introduction

Physical activity (PA) is a vital component of a healthy lifestyle and prevents morbidity and the progression of various chronic illnesses, such as obesity, diabetes, and cardiovascular disease [1,2,3,4]. Participation in regular PA is associated with significant improvements in physiological, metabolic, cognitive, and mental outcomes [5,6]. Based on the universal recognition of the associated health benefits of PA, various national and international organizations have developed standardized PA recommendations for populations and individuals to support them in achieving acceptable PA levels [7].

Among these guidelines is the World Health Organization’s (WHO) frequently cited recommendation that adults achieve at least 150 min of vigorous PA and/or 300 min of moderate/aerobic PA per week or a combination of both [8]. Nevertheless, a considerable proportion of the Saudi population does not meet these respective guidelines, and Saudi Arabia (KSA) ranks among the countries with the highest physical inactivity levels worldwide [9,10]. In a 2020 national survey, the prevalence of physical inactivity (PIA) in KSA was 71.70% among the adult male population and 91.10% among females [11]. A lack of time and support facilities were among the primary identified barriers to an active lifestyle in the Saudi population [11,12]. An increasing prevalence of non-communicable diseases with lifestyle-related and associated morbidity and mortality in the kingdom means counteracting measures are urgently needed [13,14].

Regarding counteracting strategies, a broad body of research supports PA counseling and physical education (PE) as an effective component of behavior change support in the general population [15,16,17,18]. PA counseling has been shown to effectively trigger positive behavioral and biomedical adaptations [15,16,17,18]. For example, a meta-analysis study of 15,269 adults showed that PA counseling positively affected the usual care group (*p* = 0.008) and increased self-reported PA [19]. In addition, a 6-month PA counseling intervention for hypertensive patients in Taiwan (*n* = 202) helped participants reduce their systolic blood pressure by seven mmHg (95% CI, −11.5 to −2.5 mmHg, *p* = 0.002) [20]. In South Korea (*n* = 85), a combination of PA counseling programs and dietary intervention resulted in a significant reduction in systolic and diastolic blood pressure (134 mmHg; 95% confidence interval [CI], 131 to 137; *p* = 0.011) among people with prehypertension and mild hypertension [21]. As a result of this evidence, many other countries, such as Germany, Brazil, and Finland, have proactively began advocating for and incorporating PA counseling into health-promoting programs across various clusters of their population [15,22,23]. Similar to other countries, KSA also recognized the need for PA promoting initiatives in its population. Over the past decade, several comprehensive efforts have been put into practice, ranging from school-related programs and frequent mass participation events to initiatives targeting PA promotion in clinical populations [24]. The latest governmental program was the “Saudi Vision 2030” which includes a healthy lifestyle promotion program targeting the entire Saudi population [25].

Despite an apparent need to better understand the situation of clinical PA promotion standards and related efficacy and implementation problems in KSA, more systematically gathered and structured information is needed. In addition, local clinicians and researchers frequently raised concerns about the insufficient availability of PA promotion and counseling for students and various population groups in KSA [11,12]. Therefore, the purpose of this study was to systematically compile information about the availability, characteristics, and outcomes of current PA-promoting initiatives, as well as counseling clinicians’ perceived implementation barriers to promoting the understanding of this vital intervention function within the context of chronic care management. 

## 2. Materials and Methods

We conducted a systematic literature search of four online databases: Web of Science, PubMed, ScienceDirect, and The Cochrane Library. The Preferred Reporting Items for Systematic Review and Meta-Analyses (PRISMA) criteria were used to conduct this study. The search terms used were PA counseling, promotion, PA intervention, and Saudi Arabia. Only studies conducted in adult patient populations were considered. The word “AND” was used to combine search terms, and after articles were retrieved, references were examined for more information and completed in consultation with several experts. We included studies if they met the following criteria: (1) studies focused on the prevalence of routine PA counseling in KSA, (2) PA or exercise prescription from healthcare providers, (3) PA intervention or exercise program, (5) published in the English language, (6) quantitative or qualitative studies. Studies not carried out in KSA were excluded. Two researchers thoroughly evaluated and independently assessed all selected article titles and abstracts in compliance with the inclusion criteria.

**Figure 1:**
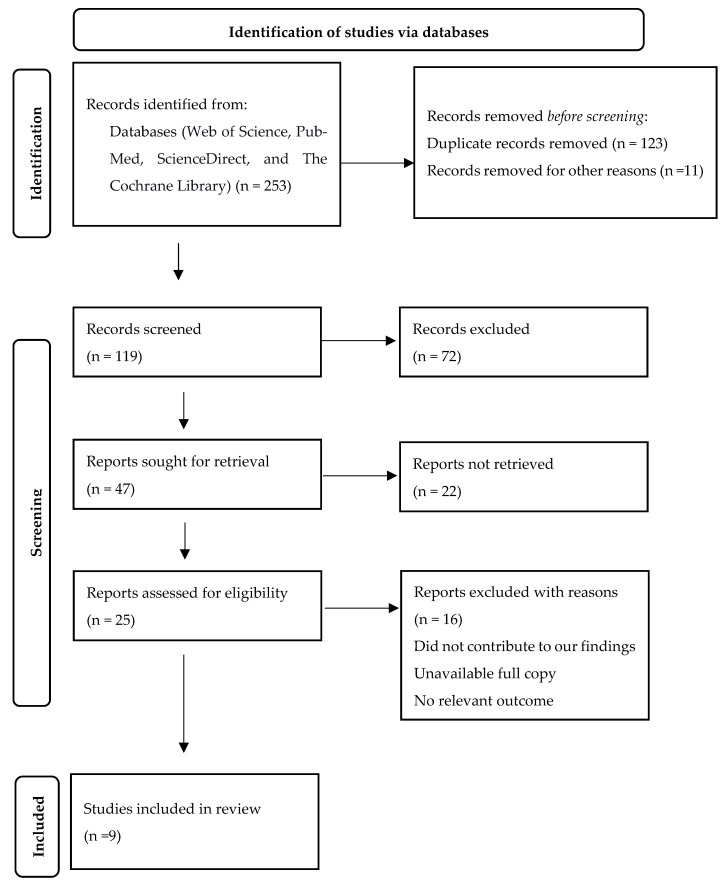
Flow diagram for the selection of studies included in a systematic review of physical activity counseling in KSA.

After the preliminary screening, a specialist analyzed the included studies and reviewed them for significance and potential duplication. Details about the author, location of the study, sample, and major findings were extracted from each study that met the inclusion criteria. Data selection and extraction are presented in Figure 1. Each study was assessed and evaluated using the Mixed Methods Appraisal Tool (MMAT) for methodological quality. Each study was rated from 25% to 100% based on its methodological design and compliance with all criteria and guidelines.

## 3. Results

Detailed information about the literature review process is presented in Figure 1. The initial search yielded 253 results. Following the removal of duplicates, 119 titles and abstracts were screened, and 47 studies were reviewed for full-text screening and further examination. In total, 25 articles were assessed for eligibility and subjected to exhaustive examination. After a detailed assessment, only nine studies met the inclusion criteria. The examined studies were quantitative studies that captured descriptive and numerical data on PA counseling. Four were cross-sectional, three were experimental, and two were interventional studies. Primary care and family medicine physicians and nurses were among the providers in the included studies. All patients in the included studies primarily suffered from chronic conditions, such as coronary heart disease, hypertension, type 2 diabetes, depression, and obesity. Table 1 presents the characteristics and outcomes of included studies.

### 3.1. PA Promotion in the Clinical Context: Characteristics, Content, and Components

Four studies described information (such as structure, content, etc.) about clinical PA promotion interventions in KSA or the implementation of PA counseling as a prescription in the context of lifestyle-related treatment protocols [25,27,28,31]. 

### 3.2. Intervention Studies

Alfawaz et al. (2019) [27] conducted an experimental study testing the impact of one-time general lifestyle advice compared to a monitored and structured 6-month lifestyle modification program with glycemic, micronutrient, and mineral indices in 160 Saudis with prediabetes. In the intervention group, the PA counseling component included knowledge transfer and the encouragement of exercising 20–30 min at least three times a week through various activities (e.g., running and jogging) [27]. PA information was also provided with written and visual information material. Meanwhile, the control group only received one-time general information about the risk factors associated with prediabetes. Khouja et al. (2020) [28] published the findings of an experimental study conducted in 2015 that compared the effects of a three-month intervention involving lifestyle modification and PA increases with standard care in Saudi females (≥30 years) with a moderate to high risk of cardiovascular disease (CVD) in the western city of Jeddah (*n* = 59). PA counseling was only available to the women in the intervention group (*n* = 31) and consisted of general health education and morning or afternoon exercise training delivered individually or in groups. The intervention lasted one month and was inspired by the U.S. Department of Health and Human Services “Be Active Your Way” program and the Saudi Ministry of Health’s National Guidelines for the Management of Cardiometabolic Risk Factors. The PA counseling and training goal was to teach individuals how to fit a daily 30-min moderate-intensity PA into their schedule.

In 2011, Midhet and Sharaf conducted an uncontrolled experimental study that assessed the impact of health education on diet, smoking, and exercise among patients with chronic diseases from 18 primary healthcare centers in the Al Qassim Region in KSA (*n* = 1254) [25,31]. During the 6-month intervention, patients received health education in the PHC from doctors and health educators who received previous training related to their communication and health education practice abilities. Using leaflets, booklets, and charts provided by the Ministry of Health, health education among attending patients focused on the risks of physical inactivity, a poor diet, and smoking [25,31]. 

### 3.3. Observational Studies

In their questionnaire-based cross-sectional study, Midhet and Al-Mohaimeed (2012) aimed to determine the impact of indoor education on chronic patients’ dietary habits and PA following discharge from a secondary hospital in the Qassim region (*n* = 169) [30]. Health education information was retrieved retrospectively from the patient file and the patient’s self-report. Key intervention characteristics were the session type (individual vs. with the family), the number of sessions during a hospital stay (none up to two or more), and additional advice provided by the treating physician.

Although patient file information differed from the patient self-report, most received individual health counseling on three occasions (30%), largely without family inclusion (58%). In Al-Ghamdi et al.’s (2018) observational study (*n* = 803), different healthcare professionals were asked to indicate which advice and recommendations were given to healthy and chronically ill patients, respectively [29]. The physical and psychological benefits of PA and the intensity (low, moderate, or vigorous) and type of exercise were addressed during the consultation. Generally, walking was the most recommended exercise for apparently healthy individuals, followed by other forms of aerobic exercise, such as swimming or running. For the chronically ill, significantly more advice was given to obese patients compared to other disease groups. 

### 3.4. Knowledge, Attitudes, and Physicians’ Perceived Competence

Four studies assessed physicians’ knowledge, attitudes, and practices regarding PA counseling [26,29,32,33]. Alahmed et al.’s (2019) cross-sectional study (with convenience sampling) investigated the PA knowledge, attitudes, and practices of primary health care physicians in KSA (*n* = 147) and found that the majority of the clinicians perceived PA promotion within their scope of responsibilities (59%) [26]. Furthermore, more than half of their participants (53%) reported high levels of perceived competence to promote PA (i.e., feeling capable of successfully guiding PA), although only 21% shared they had received training about PA counseling during medical school. When examined, nearly 60% of the participants demonstrated poor knowledge of recognized PA guidelines. Still, a high self-efficacy perception toward PA promotion was perceived by 58% of the participants. Al-Ghamdi et al. (2018) reported high perceived competence in healthcare professionals [29]. The majority of the 803 clinicians (e.g., physicians, nurses, and health educators) thought they had enough knowledge to advise patients on PA adequately [29]. A similar result was also found in a cross-sectional study by Al Shammari et al. (2016) [32] which examined the extent to which doctors proactively provided patient advice regarding the importance of PA. The results indicated that PA was an essential factor of advising patients with chronic illness [32]. Another cross-sectional study by AlRashdi et al. (2015) also found that physicians positively promoted PA counseling [33]. The majority of the physicians considered themselves effective PA promoters. 

In addition, three studies found various factors influencing physicians’ attitudes [29,32,33]. Alahmed et al. (2019) reported that female physicians were more likely to provide PA counseling than male physicians [26]. Additionally, general practitioners and internationally educated physicians were more likely to provide PA counseling to their patients than specialists and those with a local degree [29]. Furthermore, patients without chronic disease were more likely to be referred to PA examination and care by younger physicians. In a study by Al Shammari et al. (2016), family physicians reported that they counselled their patients about physical activity, especially if the patient has diabetes [32]. Finally, in a study by AlRashdi et al. (2015), most primary healthcare physicians reported advising patients to increase PA, and most considered themselves effective health promoters [29].

### 3.5. Barriers to PA Counseling

Three studies revealed barriers to routine PA counseling by physicians in KSA [26,29,32]. The most frequently reported barrier was a lack of time for PA discussion with patients, followed by a lack of training in PA counseling, and a lack of patient cooperation [26,29]. Other reported barriers included insufficient educational materials for patients and a lack of financial incentives [33]. 

### 3.6. Reported Outcomes and Efficacy of PA Counseling

The effects of PA counseling or the prescription of PA were assessed in four studies [25,27,28,31]. Alfawaz (2019), for example, discovered a significant increase in PA levels after a 6-month interventional modification program (*n* = 80, *p* < 0.05) [27]. Participants with prediabetes (20–60 years old) raised moderate physical exercise from 2.3 to 2.6 times per week after six months of intervention and counseling. Furthermore, Alfawaz (2019) [27] discovered significant differences in vigorous PA per week from 0.40 times to 1.40 times per week between the control and counseling groups. The counseling group of prediabetic patients outperformed the control group. The effect of a community-based lifestyle program that focused on PA levels and clinical outcomes for women aged 30 years old and above was assessed by Khouja et al. (2020) [25]. The researchers detected a significant reduction in systolic blood pressure (−9.2 mmHg; *p* = 0.09), blood glucose (−45 mg/dL; *p* = 0.03), and Framingham risk score (−13.6; *p* < 0.01). Finally, two studies found that a health education program significantly affected the health behaviors, such as smoking, diet, and exercise routines (e.g., brisk walks and football), of chronic disease patients attending primary health care clinics in KSA [25,31].

## 4. Discussion

After an extensive literature search, we identified studies focusing on clinical PA counseling in KSA. In total, nine were eligible and provided insight into the delivered content, the perceived competencies of healthcare providers conducting PA counseling, the barriers or issues affecting routine practice, and the outcomes and efficacy of local PA counseling studies. Documentation of this type of PA promotion method still needs to be improved in KSA, as evidenced by the small number of eligible studies.

Our results revealed that most studies used PA counseling as a component of intervention trials [25,27,28]. Besides counseling for PA and diet, interventions frequently contained theoretical health education components and behavior-based intervention functions, such as exercise training sessions. Regarding PA counseling content, the most prevalent topics were how to increase personal PA levels through different exercises and recommendations related to respective exercise duration (e.g., 20–30 min of exercise for various activities, such as running and jogging, at least three times a week). This suggests that PA counseling in clinical intervention studies has piqued the interest of researchers and local clinicians and that, as in other countries [7,34], various components are used in local PA counseling protocols.

Regarding the efficacy of PA counseling in KSA, our results indicate a significant impact of counseling measures on behavioral and biomedical outcomes in the study populations. However, eligible studies were inhomogeneous and strongly differed in their design and duration, which inhibited reliable conclusions concerning the most effective implementation approaches. More studies with standardized designs are needed to evaluate the efficacy and, on a larger scale, the efficaciousness of PA counseling interventions in KSA.

A treating clinician’s positive attitude and PA-related competencies have significantly impacted PA-promoting efforts [35]. Our findings suggest that many Saudi Arabian healthcare providers, particularly female family physicians, affect PA counseling positively [26]. Enthusiasm and a high perceived self-efficacy in successfully promoting PA in patients were prevalent in the investigated clinician populations. However, insufficient knowledge about PA guidelines and recommendations was seen as a challenge among the participating physicians [27]. A lack of PA-related knowledge and competence as a potential barrier to effective PA counseling has also been reported in other countries [36,37,38]. 

In accordance with the opinions of other researchers [9], we assume that insufficient knowledge of PA counseling may decrease the likelihood of physicians to incorporate PA counseling in their treatment approaches and may compromise positive attitudes toward it.

We suggest promising intervention opportunities to address this issue and increase PA-related knowledge and counseling competence in Saudi clinicians, such as the standardized provision of educational materials and scheduling a follow-up visit with a patient after counseling. We consider practice-oriented postgraduate PA counseling curriculums, short training programs, and regular refresher courses most appropriate for cultural and community-specific prerequisites. 

Despite knowledge transfer, such education courses should provide clinicians with concrete, evidence-based, and culturally sensitive implementation strategies for uncomplicated use in clinical practice. One notable strategy is the Five A’s (Assess, Advise, Agree, Assist, and Arrange) framework [39,40]. In this strategic approach, the healthcare provider assesses the patient’s current PA level before advising the patient via counseling messages [39,40]. Following that, the clinician and patient agree on a specific action plan in a collaborative decision-making process. Finally, the clinician contributes to the patient’s behavioral changes by providing educational materials and scheduling a follow-up visit. Peterson et al. found that this intervention (following the Five A’s framework) alleviated the lack of time for PA counseling [41].

In addition to a lack of knowledge, our results also highlighted a lack of time within standard counseling and patient cooperation as the most frequent barriers for clinicians [42,43,44]. We believe that to tackle the inactivity challenge through PA counseling programs, clinicians, researchers, and policymakers in KSA need to have a sound understanding of the barriers local clinicians commonly face. Our results suggest that a conglomerate of interpersonal and environmental factors might inhibit standardized and compelling PA counseling in KSA. On the side of physician and environmental preconditions (e.g., the clinical environment), measures to increase perceived competence (i.e., capacity building in the healthcare workforce) and targeted changes concerning time-management and treatment components in standard clinical care are assumed to create a growth in positive attitudes and more frequent incorporation of PA counseling within routine patient care. In addition, more efforts are needed to resolve the provision of educational materials and scheduling follow-up visits to impart the knowledge and benefits of PA. Healthcare providers and clinicians in KSA have an essential role in promoting PA, and they will need tools to establish and encourage PA in various populations. 

On the patient’s side, an evidence-based understanding of the reasons behind the lack of cooperation and/or compliance with physician recommendations is needed. Recent investigations into the psycho-social determinants of PA behavior among Saudi patients with non-communicable diseases indicated that a lack of motivational quality was negatively associated with PA behavior [45,46]. That lack of family support suggested a relationship with inactivity [45]. Empirical knowledge of common behavioral determinants in patients will aid clinicians to better understand patient behavior (i.e., patients’ reasons for incompliance) and address respective hurdles in a targeted manner. 

If the goal is to increase the patient’s motivation to adhere to PA protocols, specific “best practice” recommendations for interventions are required. One relevant opportunity is the smart and strategic inclusion of lifestyle-related patient care into large-scale government community initiatives. For example, the objectives of Vision 2030 are to increase public participation in PA and sports and strengthen the prevention of health threats. Under this umbrella, various mass-participation initiatives for PA promotion are implemented, which offer the potential for anchoring healthcare-specific initiatives [24]. Government-backed, large-scale programs in other countries have shown the potential of connecting lifestyle-related patient care with community-based interventions [47].

We believe that such intervention strategies—if matched to cultural and community-specific needs and preconditions—have considerable potential to increase PA adherence in clinical populations in KSA. We encourage policymakers to invest in developing empirically informed programs that intelligently link mass participation initiatives with standard patient care.

Furthermore, we recommend incorporating PA-promoting interventions in the clinical setting in long-term projects that encourage interdisciplinary collaboration and communication between different clinicians (e.g., doctors, nurses, physiotherapists, etc.) to achieve adequate PA levels in the respective patient population.

Finally, we would like to encourage hospital guidelines and treatment standards to be revised so that “exercise as prescription,” in conjunction with an associated counseling practice, is actively promoted for all patient groups who do not have contraindications to PA.

This investigation has some limitations. First, the small number of studies included may not be able to assess the program structures of PA counseling in KSA fully. Second, many papers evaluated in this study were cross-sectional studies which cannot be used to establish causality. Third, the small sample sizes and sampling strategies used in some of the included studies may limit the generalizability of the findings. 

## 5. Conclusions

In conclusion, this review provides an overview of the characteristics, outcomes, and perceived barriers of PA counseling in KSA. Data show that PA counseling in KSA focuses on and contains health education components and behavior-based intervention functions. In addition, PA counseling delivered by healthcare professionals and clinicians appears to effectively increase the PA levels of participants and decrease clinical characteristics. Furthermore, female family physicians are more likely to promote PA than male physicians. A lack of time for PA discussion with the patients, a lack of training in PA counseling, and a lack of patient cooperation are considered major barriers to PA counseling. It has been demonstrated that there is currently relatively little structured evidence for PA counseling in KSA, especially for this health promotion technique as part of standard patient care, as opposed to experimental settings. Many Saudi healthcare providers, particularly female family physicians, seem to promote PA enthusiastically and self-confidently within their treatment approaches. Self-perceived responsibility in the PA advisory function and high perceived competence also seem to be common among local clinicians. The results further indicate that demographic and educational factors influence the attitudes of PA counseling clinicians and that specific barriers, largely of a timely and education-related nature, might inhibit its implementation. Various tools, such as the internet and YouTube, may help patients’ compliance and increase the effect of PA in adults and children. Large-scale studies are required to determine the prevalence of PA counseling, related barriers, the corresponding characteristics in KSA, and the efficacy of various approaches. This review can add valuable information and may serve as a guideline among clinicians and health policymakers to develop a PA counseling program. In addition, the review could be used to overcome barriers to the implementation of PA counseling in KSA. Despite the small number of studies included, this review contributes to the limited understanding of current PA counseling practices in Saudi Arabia and serves as a starting point for further research.

## Figures and Tables

**Table 1:** Characteristics and outcomes of included studies.

	Author and Year	Methodological Appraisal Score	Participant	Region/City	Program/Training	Outcome
1	Alahmed et al., 2019 [26]	75	147 physicians	Eastern Province, SA (Al-Khobar, Dammam, Qatif, and Safwa)	KAP study of PA counseling	Only 21.8% had received training about PA counseling during medical school or their residency program. Almost 60% of physicians believed that PA promotion to patients was their responsibility and felt confident in their ability to provide PA counseling. Lack of time, inadequate referral services for PA, and inadequate training in PA counseling were considered barriers to providing PA counseling.
2	Alfawaz et al., 2019 [27]	75	160 Saudis with prediabetes	Riyadh	Two-armed lifestyle modification program	A significant improvement was found in the glycemic indices of the intervention group after a 6-month lifestyle modification program. Additionally, significant improvements in dietary habits and physical activity levels were more apparent in the intervention group than the control group.
3	Khouja et al., 2020 [28]	75	Women ≥30 years old with a moderate to high risk of CVD	Jeddah	Three-month intervention involving lifestyle modification and physical activity with standard care	The lifestyle intervention program significantly reduced systolic blood pressure (−9.2 mmHg), blood glucose (−45 mg/dL), and Framingham risk score (−13.6).
4	Al-Ghamdi et al., 2018 [29]	75	803 healthcare providers	Riyadh	KAP study of PA counseling	A significant difference in the opinions of healthcare professionals regarding their perceptions of PA levels among the general population was observed. The data showed that most primary care staff were quite enthusiastic about promoting physical activity among their patients and revealed that they routinely discussed and advised their patients about the benefits of physical fitness. However, there were some factors that acted as barriers to promoting PA, such as lack of time, lack of educational materials for patients, lack of proper training and protocols, lack of patient cooperation, and lack of financial incentive.
5	Midhet (2013) [30]	75	169 patients	Al Qasim	Patient health education	Health education sessions were significantly associated with healthier diet and regular exercise of patients.
6	Midhet (2011) [31]	75	Baseline (*n* = 1254) and follow-up (*n* = 1011) attendees in PHCC centers	Al Qasim	Trained the PHCC staff in health education skills and introduced health education seminars	Compared to the baseline, male respondents in the follow-up survey were less likely to smoke and more likely to do regular exercise (such as doing brisk walks and playing football).
7	Sharaf (2011) [25]	75	Patients with chronic diseases (e.g., coronary artery disease)	Al Qassim Region	The intervention included refresher training for PHC centers’ staff to improve communication skills and the use of health education materials	Among chronic disease patients, significant improvements in smoking, diet, and exercise habits were observed at the end-line survey compared to the baseline.
8	Al Shammari (2016) [32]	75	80 family medicine residents	Al Khobar	KAP study of PA counseling	The majority of the participants counseled their patients about physical activity, especially if the patient had diabetes. Residents of the joint family medicine program of the eastern province of the KSA self-reported that while they were not physically active themselves, they were active in in-patient counseling regarding the importance of physical activity to achieve global health and well-being.
9	AlRashdi (2015) [33]	75	80 primary care physicians	Riyadh	Attitudes and perceived = barriers of primary care physicians	Advice to increase physical activity was given to patients by most participants, and most considered themselves effective health promoters. Lack of time, patients ignoring physician recommendations, and insufficient educational materials for patients were the most frequently perceived barriers by primary care physicians regarding promoting physical activity.

## Data Availability

Not applicable.

## References

[1] Booth F.W., Roberts C.K., Laye M.J. (2012). Lack of exercise is a major cause of chronic diseases. Compr. Physiol..

[2] Nieman D.C., Wentz L.M. (2019). The compelling link between physical activity and the body’s defense system. J. Sport Health Sci..

[3] da Silveira M.P., da Silva Fagundes K.K., Bizuti M.R., Starck É., Rossi R.C., de Resende e Silva D.T. (2021). Physical exercise as a tool to help the immune system against COVID-19: An integrative review of the current literature. Clin. Exp. Med..

[4] Reiner M., Niermann C., Jekauc D., Woll A. (2013). Long-term health benefits of physical activity—A systematic review of longitudinal studies. BMC Public Health.

[5] Cerletti P., Keidel D., Imboden M., Schindler C., Probst-Hensch N. (2020). The modifying role of physical activity in the cross-sectional and longitudinal association of health-related quality of life with physiological functioning-based latent classes and metabolic syndrome. Health Qual. Life Outcomes.

[6] Mandolesi L., Polverino A., Montuori S., Foti F., Ferraioli G., Sorrentino P., Sorrentino G. (2018). Effects of Physical Exercise on Cognitive Functioning and Wellbeing: Biological and Psychological Benefits. Front. Psychol..

[7] Pfeifer R.A., Aljuraiban G.S., AlMarzooqi M.A., Alghannam A.F., BaHammam A.S., Dobia A.M., Alothman S.A., Aljuhani O., Aljaloud K.S. (2021). The recommended amount of physical activity, sedentary behavior, and sleep duration for healthy Saudis: A joint consensus statement of the Saudi Public Health Authority. Ann. Thorac. Med..

[8] World Health Organization (2020). WHO Guidelines on Physical Activity and Sedentary Behavior. https://www.who.int/publications/i/item/9789240015128.

[9] Alahmed Z., Lobelo F. (2018). Physical activity promotion in Saudi Arabia: A critical role for clinicians and the health care system. J. Epidemiol. Glob. Health.

[10] Mabry R., Koohsari M.J., Bull F., Owen N. (2016). A systematic review of physical activity and sedentary behaviour research in the oil-producing countries of the Arabian Peninsula. BMC Public Health.

[11] Alqahtani B.A., Alenazi A.M., Alhowimel A.S., Elnaggar R.K. (2021). The descriptive pattern of physical activity in Saudi Arabia: Analysis of national survey data. Int. Health.

[12] Samara A., Nistrup A., Al-Rammah T.Y., Aro A.R. (2015). Lack of facilities rather than sociocultural factors as the primary barrier to physical activity among female Saudi university students. Int. J. Womens Health.

[13] Herzallah H.K., Antonisamy B.R., Shafee M.H., Al-Otaibi S.T. (2019). Temporal trends in the incidence and demographics of cancers, communicable diseases, and non-communicable diseases in Saudi Arabia over the last decade. Saudi Med. J..

[14] The Institute for Health Metrics and Evaluation (IHME) (2019). Saudi Arabia. http://www.healthdata.org/saudi-arabia.

[15] Florindo A.A., Mielke G.I., de Oliveira Gomes G.A., Ramos L.R., Bracco M.M., Parra D.C., Simoes E.J., Lobelo F., Hallal P.C. (2013). Physical activity counseling in primary health care in Brazil: A national study on prevalence and associated factors. BMC Public Health.

[16] Wattanapisit A., Tuangratananon T., Thanamee S. (2018). Physical activity counseling in primary care and family medicine residency training: A systematic review. BMC Med. Educ..

[17] Sousa Junior A.E., Macêdo G.A.D., Schwade D., Sócrates J., Alves J.W., Farias-Junior L.F., Freire Y.A., Lemos T.M.A.M., Browne R.A.V., Costa E.C. (2020). Physical Activity Counseling for Adults with Hypertension: A Randomized Controlled Pilot Trial. Int. J. Environ. Res. Public Health.

[18] Lin J.S., O’Connor E.A., Evans C.V., Senger C.A., Rowland M.G., Groom H.C. (2014). Behavioral Counseling to Promote a Healthy Lifestyle for Cardiovascular Disease Prevention in Persons with Cardiovascular Risk Factors: An Updated Systematic Evidence Review for the U.S. Preventive Services Task Force.

[19] Oloo M.O., Wamukoya E.K., Wanzala M. (2020). Efficacy of Physical Activity Counselling Interventions Delivered in Primary Care: A Systematic Review And Meta-Analysis. Eur. J. Phys. Educ. Sport Sci..

[20] Lee L.L., Arthur A., Avis M. (2007). Evaluating a community-based walking intervention for hypertensive older people in Taiwan: A randomized controlled trial. Prev. Med..

[21] Lee C.J., Kim J.Y., Shim E., Hong S.H., Lee M., Jeon J.Y., Park S. (2018). The Effects of Diet Alone or in Combination with Exercise in Patients with Prehypertension and Hypertension: A Randomized Controlled Trial. Korean Circ. J..

[22] Füzéki E., Weber T., Groneberg D.A., Banzer W. (2020). Physical Activity Counseling in Primary Care in Germany—An Integrative Review. Int. J. Environ. Res. Public Health.

[23] Rasinaho M., Hirvensalo M., Törmäkangas T., Leinonen R., Lintunen T., Rantanen T. (2012). Effect of physical activity counseling on physical activity of older people in Finland (ISRCTN 07330512). Health Promot. Int..

[24] Al-Hazzaa H.M., AlMarzooqi M.A. (2018). Descriptive Analysis of Physical Activity Initiatives for Health Promotion in Saudi Arabia. Front. Public Health.

[25] Sharaf F. (2010). Impact of health education on compliance among patients of chronic diseases in Al Qassim, Saudi Arabia. Int. J. Health Sci..

[26] Alahmed Z., Lobelo F. (2019). Correlates of physical activity counseling provided by physicians: A cross-sectional study in Eastern Province, Saudi Arabia. PLoS ONE.

[27] Alfawaz H., Naeef A.F., Wani K., Khattak M.N.K., Sabico S., Alnaami A.M., Al-Daghri N.M. (2019). Improvements in Glycemic, Micronutrient, and Mineral Indices in Arab Adults with Pre-Diabetes Post-Lifestyle Modification Program. Nutrients.

[28] Khouja J.H., Al Jasir B., Bargawi A.A., Kutbi M. (2020). Lifestyle Intervention for Cardiovascular Disease Risk Factors in Jeddah. Saudi Arabia. Cureus.

[29] Al-Ghamdi S., Alajmi M., Al-Gonaim A., Al-Juhayyim S., Al-Qasem S., Al-Tamimi I. (2018). Perceptions and attitudes of primary healthcare providers in Riyadh City, Saudi Arabia, toward the promotion of physical activity. Int. J. Health Promot. Educ..

[30] Midhet F.M., Al-Mohaimeed A. (2013). Impact of indoor education on the lifestyles of patients with chronic disease in a secondary hospital in Qassim, Kingdom of Saudi Arabia. J. Taibah Univ. Med. Sci..

[31] Midhet F.M., Sharaf F.K. (2011). Impact of health education on lifestyles in central Saudi Arabia. Saudi Med. J..

[32] Al Shammari M. (2016). Are Family Medicine Residents Physically Active? And Do They Counsel Their Chronically Ill Patients about Physical Activity? A Cross-Sectional Study among Residents of the Family Medicine Joint Program, Eastern Province, Saudi Arabia. Int. J. Med. Sci. Public Health.

[33] Al Rashidi M. (2018). Attitudes and Barriers of Primary Care Physicians toward Promoting Physical Activity to Patients in Prince Sultan Military Medical City, Riyadh, Saudi Arabia. Saudi J. Oral Dent. Res. (SJODR).

[34] Geidl W., Wais J., Fangmann C., Demisse E., Pfeifer K., Sudeck G. (2019). Physical activity promotion in daily exercise therapy: The perspectives of exercise therapists in German rehabilitation settings. BMC Sports Sci. Med. Rehabil..

[35] Gnanendran A., Pyne D.B., Fallon K.E., Fricker P.A. (2011). Attitudes of medical students, clinicians and sports scientists towards exercise counselling. J. Sports Sci. Med..

[36] Solmundson K., Koehle M., McKenzie D. (2016). Are we adequately preparing the next generation of physicians to prescribe exercise as prevention and treatment? Residents express the desire for more training in exercise prescription. Can. Med. Educ. J..

[37] Hébert E.T., Caughy M.O., Shuval K. (2012). Primary care providers’ perceptions of physical activity counseling in a clinical setting: A systematic review. Br. J. Sports Med..

[38] Huijg J.M., Gebhardt W.A., Verheijden M.W., Phillips E.M. (2015). Factors influencing primary health care professionals’ physical activity promotion behaviors: A systematic review. Int. J. Behav. Med..

[39] Estabrooks P.A., Glasgow R.E., Dzewaltowski D.A. (2003). Physical activity promotion through primary care. JAMA.

[40] Meriwether R.A., Lee J.A., Lafleur A.S., Wiseman P. (2008). Physical activity counseling. Am. Fam. Physician.

[41] Peterson J.A. (2007). Get moving! Physical activity counseling in primary care. J. Am. Acad. Nurse Pract..

[42] Antognoli E.L., Seeholzer E.L., Gullett H., Jackson B., Smith S., Flocke S.A. (2017). Primary care resident training for obesity, nutrition, and physical activity counseling: A mixed-methods study. Health Promot. Pract..

[43] Malatskey L., Bar Zeev Y., Tzuk-Onn A., Polak R. (2017). Lifestyle medicine course for family medicine residents: Preliminary assessment of the impact on knowledge, attitudes, self-efficacy and personal health. Postgrad. Med. J..

[44] Omura J.D., Bellissimo M.P., Watson K.B., Loustalot F., Fulton J.E., Carlson S.A. (2018). Primary care providers’ physical activity counseling and referral practices and barriers for cardiovascular disease prevention. Prev. Med..

[45] Saller F.V. (2021). Qualitative Analysis of Psycho-Social Factors of Potential Influence on Physical Activity and Dietary Practice of Patients with Diabetes and Cardiovascular Disease in Saudi Arabia. Saudi J. Nurs. Health Care.

[46] Saller F.V., Mohammed A., Al Dhaferi F. (2020). Physical activity and behavioral regulations for exercise in patients with noncommunicable disease in central Saudi Arabia. Saudi J. Sports Med..

[47] Fleming J., Bryce C., Parsons J., Wellington C., Dale J. (2020). Engagement with and delivery of the ‘parkrun practice initiative’ in general practice: A mixed methods study. Br. J. Gen. Pract..

